# Environmental Sample Stability for Pharmaceutical Compound Analysis: Handling and Preservation Recommendations

**DOI:** 10.1155/2023/5526429

**Published:** 2023-10-19

**Authors:** Nuning Vita Hidayati, Laurence Asia, Stephanie Lebarillier, Ita Widowati, Agus Sabdono, Anne Piram, Rizqi Rizaldi Hidayat, Dina Fitriyah, Indra Putra Almanar, Pierre Doumenq, Agung Dhamar Syakti

**Affiliations:** ^1^Fisheries and Marine Sciences Faculty, Jenderal Soedirman University, Kampus Karangwangkal, Jl. Dr. Suparno, Purwokerto 53123, Indonesia; ^2^Center for Maritime Biosciences Studies, Institute for Research and Community Service, Jenderal Soedirman University, Kampus Karangwangkal, Jl. Dr. Suparno, Purwokerto 53123, Indonesia; ^3^Aix Marseille University, CNRS, LCE, Marseille, France; ^4^Faculty of Fisheries and Marine Sciences, Universitas Diponegoro, Jl. Prof. Soedharto, SH, Tembalang, Semarang 50275, Indonesia; ^5^Maritime Technique and Technology Faculty, Raja Ali Haji Maritime University, Jl. Politeknik Senggarang, Tanjungpinang, Riau Islands 29100, Indonesia; ^6^Marine Sciences and Fisheries Faculty, Raja Ali Haji Maritime University, Jl. Politeknik Senggarang, Tanjungpinang, Riau Islands 29100, Indonesia

## Abstract

Efficient and resilient techniques for handling samples are essential for detecting pharmaceutical compounds in the environment. This study explores a method for preserving water samples during transport before quantitative analysis. The study investigates the stability of 17*α*-ethynylestradiol (EE2), acetaminophen (ACM), oxytetracycline (OTC), sulfamethoxazole (SMX), and trimethoprim (TMP) after preconcentration within solid-phase extraction (SPE) cartridges. Through various experiments involving different holding times and storage temperatures, it was determined that four pharmaceutical compounds remained stable when stored for a month at 4°C and for six months when stored at −18°C in darkness. Storing these compounds in SPE cartridges at −18°C seemed effective in preserving them for extended periods. In addition, ACM, TMP, OTC, EE2, and SMX remained stable for three days at room temperature. These findings establish guidelines for appropriate storage and handling practices of pharmaceutical compounds preconcentrated from aqueous environmental samples using SPE.

## 1. Introduction

The emergence of pharmaceuticals within the environment has become a topic of heightened public concern due to their potential ecological ramifications. These pharmaceutical compounds find their way into the environment through diverse avenues such as wastewater treatment plant discharge, improper disposal of expired medications [1–4], pharmaceutical industry and hospital effluents [5], and animal waste discharge into the environment. The ubiquity of pharmaceuticals, including acetaminophen (ACM), oxytetracycline (OTC), sulfamethoxazole (SMX), trimethoprim (TMP), and 17*α*-ethynylestradiol (EE2), has raised concerns regarding their environmental impact [6–8]. In the context of this study, these selected pharmaceuticals hold particular significance as they are currently priority targets in Indonesia's efforts to detect their presence in aquatic environments and assess associated environmental risks [9–12].

ACM, or paracetamol, is a globally widespread antipyretic and analgesic, often used as a primary treatment for headaches, including migraines, a significant contributor to disability, particularly among young women [13–16]. ACM's availability without prescription and its affordability in the Indonesian context has led to its wide utilization [17, 18]. The detection of ACM in various aquatic environments, coupled with its potential threat to aquatic organisms, warrants attention [19–22].

OTC, SMX, and TMP are widely used antibiotics for therapeutic purposes in humans, as well as in farmed aquatic animals (including fish and shrimp) to treat bacterial diseases [23, 24]. Those compounds are three of the top 10 list of generic antibiotic drugs [19]. The presence of OTC in environmental compartments has been detected worldwide [25], as well as TMP [26] and SMX [27]. Previous study conducted by [28, 29] demostrated OTC bioaccumulation in various organisms, including invertebrates and fish. OTC, TMP, and SMX are often detected in seafood products, especially shrimp [30, 31]. Those antibiotics have been reported to potentially cause negative impacts on the aquatic organisms [32, 33].

EE2 is a constituent of frequently used contraceptive pills and an endocrine disruptive substance [34]. A high risk of their occurrence in aquatic environments becomes a rise concern. Fertilization was significantly reduced in sea urchins when exposed to this substance. Morphological abnormalities were also observed in mussel and sea urchin embryos [35]. The synthetic estrogen EE2 also poses an adverse effect on the reproduction of aquatic organisms [36, 37]. The continuous use and the evident occurrence of those selected compounds in aquatic environments as well as the high potential ecological risks on aquatic organisms attracted our attention to investigate their stability.

Effective environmental studies necessitate well-structured sampling plans encompassing location, timing, and methodology for collection and analysis. Sample handling is paramount within the broader sampling strategy to ensure analysis accuracy [38, 39]. Changes in the analyte concentration during storage have been recognized as a potential source of bias that could occur prior to sample analysis [40]. Storage conditions are critical, as samples can be degraded through bacterial activity, exposure to light, and/or chemical interactions [41, 42]. Furthermore, it is important to obtain the maximum delay between the sample collection and analysis times to provide accurate and reliable data. This also includes transport during and or after field sampling and storage before analysis [43, 44]. For this reason, in this study, we investigated a set of conditions necessary to prevent or minimize errors introduced through sample handling and storage conditions before environmental sample analysis. In the current study, we estimated the stability of targeted pharmaceutical compounds (ACM, antibiotics, and steroid hormones) under various sample storage conditions and maximum holding times after their preconcentration involving solid phase extraction (SPE) cartridges. Given the focus on marine life, saltwater serves as the focal point. This choice is rooted in environmental, regulatory, and ecological considerations, acknowledging the significant influence of medicinal compounds on coastal ecosystems and public health.

The outcomes of this study hold profound relevance, particularly in regions with limited access to necessary laboratories and analytical instruments such as Indonesia, where the time span between sampling and analysis is extended. By addressing these challenges, the study aims to contribute invaluable insights to uphold data validity and reliability in environmental analysis.

## 2. Methodology

### 2.1. Chemicals

Acetaminophen (ACM), oxytetracycline (OTC), sulfamethoxazole (SMX), and trimethoprim (TMP) were provided by Sigma-Aldrich with a high purity standard quality (St. Louis, MO, USA). Water, methanol (MeOH), acetonitrile (ACN), formic acid (HCOOH) (98–100%), and high-performance liquid chromatography (HPLC)-grade solvents were purchased from Merck (Darmstadt, Germany) and Thermo Fisher Scientific (Thermo Scientific, Franklin, MA, USA). Hydrochloric acid (HCl) and sodium hydroxide (NaOH) were obtained from Sigma-Aldrich (St. Louis, MO, USA). We purchased disodium ethylenediamine tetraacetate (Na_2_EDTA) from VWR (France). Syringe filters with polytetrafluoroethylene (PTFE) membranes (0.22 *μ*m) were supplied by Thermo Fisher (Thermo Fisher, USA). Oasis hydrophilic-lipophilic balanced cartridges (HLB; 6 mL, 200 mg) for solid phase extraction (SPE) were purchased from Waters (Milford, MA, USA). A Q5 Milli-Q water purification system (Millipore, Bedford, Massachusetts, USA) was used to prepare ultrapure water with a specific resistance of 18.2 MΩ·cm. Artificial seawater (pure ocean expert reef salt; Prodibio, France) was prepared by diluting salt in ultrapure water.

### 2.2. Preparation of Standard Solutions

Individual compound standard solutions (1000 mg·L^−1^) were prepared separately by dissolving an appropriate amount of each substance in 10 mL of MeOH. The stock standard solutions were then vigorously shaken to dissolve the standard material. Individual stock solutions were finally stored at −18°C in the dark until use. Working standard solutions were prepared from individual stock solutions in 10 : 90% ACN/H_2_O at a concentration of 10 mg·L^−1^. All the working solutions were stored at −10°C. These mixtures were employed to generate calibration curves and perform recovery studies.

### 2.3. Solid Phase Extraction (SPE)

Considering the need for a “controlled experimental environment,” all experiments were conducted using an artificial seawater matrix. This decision ensured a controlled and replicable experimental context. The selection of artificial seawater aimed to establish a well-defined setting. During SPE extraction, 10 mL artificial seawater samples were processed. Each seawater sample received 100 *µ*L of a standard solution. The pH was adjusted to 7 using HCl or NaOH. A 10 mM Na_2_EDTA solution was introduced to achieve a final concentration of 10 mM EDTA in the samples. Subsequently, a standard analyte solution was added to attain a final concentration of 10 mg·L^−1^. The spiked artificial seawater samples (10 mL aliquots) underwent extraction within SPE cartridges, employing a modified version of a validated analytical method [45] with some modification. Briefly, 10 mL of the seawater samples was preconcentrated in SPE cartridges. The cartridges were conditioned by successively applying 5 mL of acetonitrile, 5 mL of ultrapure water, and 5 mL of Na_2_EDTA 10 mM with a pH of 7 at a flow rate of 2 mL/min [2]. After sample loading at a rate of 1-2 mL·min^−1^, the cartridges were pumped under vacuum and then stored at various temperatures (−18°C, 4°C, and room temperature) and holding times. Five milliliters of ACN/H_2_O (% v/v) at the selected percentage composition was used as the elution solvent.

The extracted solvent was evaporated to an extract volume of approximately 50 *µ*L under a gentle stream of nitrogen at 40°C and then transferred into an amber chromatographic vial (2 mL). The collection vial was then successively washed with 2 × 50 *µ*L of ACN and adjusted to a final volume of 1.0 mL. All reconstituted extracts were passed through PTFE (0.2 *μ*m) filtration membranes before analysis.

### 2.4. Optimization of Extraction

To achieve optimal recovery of the target compounds, a range of ultrapure ACN/H_2_O solvent compositions (% v/v) (40/60; 60/40; 80/20; 90/10; and 100/0) were systematically evaluated to facilitate the separation of the analyzed compounds from the HLB cartridges. The recovery assessment was conducted at a consistent concentration of 10 mg·L^−1^.

The extraction recovery was determined using the absolute recovery (AR) formula, as specified by the following equation [46]:

(1) $$\begin{array}{c} \text{AR}=\frac{\text{Peak area of analyte in sample}}{\text{Peak area of analyte in external sandard}}. \end{array}$$

Here, “Peak area of analyte in sample” represents the peak area of the analyte in the sample that had been spiked prior to extraction, and “Peak area of analyte in external standard” pertains to the peak area of the analyte dissolved in pure solvent. Each set of experimental conditions was replicated in triplicate during the recovery assessment. To ensure robust quality assurance, three procedural blanks consisting of 10 mL of artificial seawater were included in the analysis.

### 2.5. Chromatographic Analysis

All analyses were conducted using an ultraperformance liquid chromatography (UPLC) instrument (PerkinElmer Altus 30) paired with an ultraviolet (UV) detector. Chromatographic separation was performed using a Zorbax Eclipse XDB C_18_ column (2.1 mm × 150 mm, 3.5 *µ*m) at 45°C with HPLC-grade acetonitrile enriched with 0.1% formic acid for mobile phase A and ultrapure H_2_O with 0.1% formic acid for mobile phase B. A 5 *μ*L sample of the analytes was injected at a 0.4 mL·min^−1^ flow rate. The sample reservoir was set up at a temperature of 4°C. The elution gradient was as follows: initial time—0% A; 2 min—5% A; 13.5 min—0% and maintenance until minute 16; and 17.0 min—5% A and maintenance up to 18 min. All the analytes were measured using UV detection at wavelengths of 280 nm for ACM, 218 nm for TMP, 355 nm for OTC, 248 nm for SMX, and 200 nm for EE2.

### 2.6. Method Validation

For validation, the ICH guidelines of Q2 [40] served as the established protocol, encompassing assessments of linearity, accuracy, precision, and extraction recovery. Linearity test solutions were prepared spanning concentrations from 2 to 500 mg·L^−1^, with each predefined concentration being independently prepared in triplicate.

Linearity assessment was executed through regression analysis, yielding intercept and correlation coefficient values. Accuracy and precision were determined based on recovery percentage and relative standard deviation (RSD%), respectively, both for intraday and interday assessments. Intraday precision was gauged by analyzing six replicate samples within the same day, while interday precision was evaluated over three days with samples spiked to identical concentrations, with results expressed as RSD%. Accuracy was quantified through the relative error (RE), representing the percent deviation from the nominal concentration. According to [47], intra- and interday precision criteria can be accepted with values ≤15%. The limit of detection (LOD) and limit of quantification (LOQ) were calculated by multiplying the average concentration of ten blank replicates by 3 and 10, respectively [48]. LOD values were determined as 0.19 for ACM, 0.14 for TMP, 0.12 for OTC, 0.01 for SMX, and 0.04 for EE2. Corresponding LOQ values were established at 0.62, 0.46, 0.4, 0.04, and 0.14 for ACM, TMP, OTC, SMX, and EE2, respectively. To ensure that no pollutants were present and that no significant drift in the instrument sensitivity occurred, a blank sample and a standard at a concentration of 10 *µ*g·L^−1^ were employed. Moreover, in this study, the instrument sensitivity drift was assessed and validated considering a threshold with a 10% maximum drift [49].

### 2.7. Study of the Holding Time and Storage Temperature

After the preconcentration step, a stability study was conducted by exposing the samples, which remained in their packs (SPE cartridges), to likely exposure conditions. The SPE cartridges were then stored at different commonly adopted temperatures, i.e., frozen (−18°C), cold (4°C), and room temperature (25°C), in the dark and for various lengths of time (from day 0 up to 6 months after preconcentration).

Initial sample preparation occurred on day 0 for each study, with analysis commencing within three hours of preparation. Subsequent samples were immediately stored under designated conditions until analysis. Storage at 4°C involved refrigeration until the morning of analysis, while −20°C-stored samples were thawed overnight in the refrigerator after being removed from the freezer the previous evening. To evaluate potential losses, the target compounds extracted from the SPE cartridges were analyzed and compared with freshly prepared samples.

## 3. Results and Discussion

### 3.1. Optimal Wavelength and Retention Time

**Figure 1:**
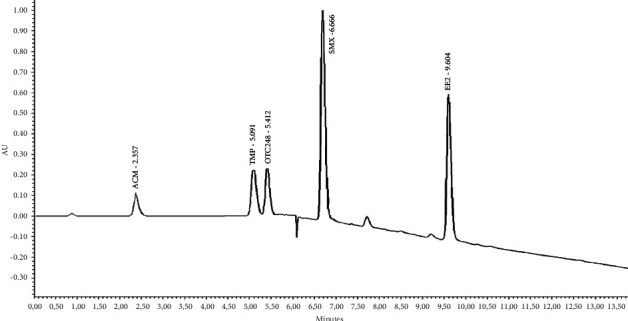
UPLC-UV chromatogram of ACM, TMP, OTC, SMX, and EE2 at selected wavelength. The retention times (RT) are 2.49 (ACM), 5.22 (TMP), 5.48 (OTC), 6.70 (SMX), and 9.6 min (EE2).

The UPLC-UV reversed phase technique was harnessed for the quantification of ACM, TMP, OTC, SMX, and EE2. To ascertain the most suitable conditions for the concurrent determination of these compounds, preliminary experiments were conducted. Different wavelengths were evaluated, leading to the identification of the most effective wavelengths for each analyte: 280 nm for ACM, 218 nm for TMP, 355 nm for OTC, 248 nm for SMX, and 200 nm for EE2. The retention times (in minutes) observed under our experimental settings were 2.49 for ACM, 5.22 for TMP, 5.48 for OTC, 6.70 for SMX, and 9.6 for EE2. A representative UPLC-UV chromatogram showcasing the selected pharmaceuticals and hormone in a mixed standard solution is depicted in Figure 1.

### 3.2. Optimization of Extraction

**Figure 2:**
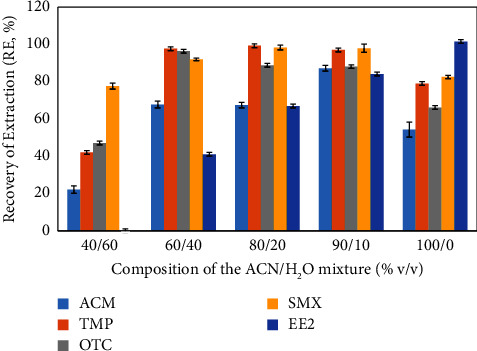
Extraction recovery for the different compositions of the elution solvent (ACN/H_2_O).

The extraction of the studied compounds was carried out using solid-phase extraction (SPE), a technique based on liquid-solid sorptive extraction through a chromatography column where analytes are selectively retained in a solid adsorbent [50]. The choice of solvent plays a crucial role in the extraction recovery. In this study, we employed acetonitrile (ACN) as the extraction solvent for ACM, SMX, TMP, OTC, and EE2 due to its proven effectiveness, especially in two-phase water pharmaceutical compound extraction. In a previous work, the authors in [51] examined methanol, ACN, ethyl acetate, and dichloromethane as sulfonamide and trimethoprim extraction solvents. Their results indicated that higher recoveries could be obtained with acetonitrile or ethyl acetate than with the other solvents. In line with these findings, we evaluated elution solutions with different ACN/H_2_O compositions: 40/60, 60/40, 80/20, 90/10, and 100/0 (% v/v). Among these compositions, 90/10 (% v/v) ACN/H_2_O provided the optimal compromise, yielding high recoveries of the targeted pharmaceuticals (Figure 2), thus being chosen for subsequent water sample analysis. SPE is a commonly used method to preconcentrate and separate drug residues [52]. Because of its versatility in terms of purification, trace preconcentration, salt elimination, derivatization, and class fractionation, SPE is suitable for sample preparation of liquid samples [53].

To retain the maximum number of target analytes and to prevent sample loss in the SPE column, the pH of the water samples must be adjusted [54]. Most of our target compounds are acidic substances that most likely exist in solution in their ionized form, which might result in poor retention in the lipophilic column, especially under neutral pH conditions [55]. The authors in [54] found that acidic groups of tetracyclines and sulfonamides were best retained in SPE columns under acidic conditions. The pKa value of these compounds might be responsible for this phenomenon. For instance, tetracycline (pKa = 3.3) and sulfamethoxazole (pKa = 5.6) have been efficiently extracted due to high retention effects in the SPE column under acidic conditions.

However, acetaminophen is a basic and neutral anti-inflammatory/analgesic drug. Thus, to ensure better retention of this group of compounds, we used Oasis lipophilic/hydrophilic balanced (HLB) packing, which is the most widely used chromatography column for the monitoring traces of basic pharmaceutical compounds.

The Oasis HLB column comprises a combination of divinylbenzene (lipophilic) and N-vinylpyrrolidone (hydrophilic) [55]. Due to the chemical composition of the Oasis HLB cartridge, it retains both polar and nonpolar emerging compounds [56]. Furthermore, using this method, a favorable performance has been achieved in the extraction of acidic to basic as well as neutral compounds.

Furthermore, in the case of tetracyclines, silanol groups might generate interference since they tend to bind irreversibly to tetracyclines. In addition, to prevent chelation of metals, a strong chelator, such as Na_2_EDTA, must be added to the sample, which is highly soluble in water [55]. The improvement involving Na_2_EDTA addition to samples to better extract pharmaceuticals, i.e., antibiotics, was suggested by Hernández, Sancho [57]. For this reason, we added a certain volume of a 100 mM Na_2_EDTA solution to the samples to ensure reproducibility and high recovery improvement for all target compounds.

### 3.3. Method Validation

Method validation was conducted according to [47] regarding the recovery, linearity, precision, and accuracy of each analyte.

### 3.4. Linearity

The observation linearity of the results was evaluated through linear regression analysis of the calibration curves within the 2–500 mg·L^−1^ range using seven calibration points. Values of the linear regression coefficient of determination *R*^2^ were calculated for each analyte with values ≥0.995, indicating a suitable linearity (*R*^2^ ≥ 0.9900).

### 3.5. Precision and Accuracy

The precision and accuracy were assessed by determining repeatability and reproducibility, respectively, throughout the experiments. Thus, repeatability (intraday precision) among the artificial seawater samples was studied at concentrations of 5, 7.5, and 10 mg·L^−1^ of each analyte, using three replicates for each selected concentration level. Reproducibility (interday precision) was evaluated using the highest concentration level of 10 mg·L^−1^, while the samples were spiked on three distinct days. Precision and accuracy were evaluated by determining the extraction recovery on three consecutive days at the following three levels: 5, 7.5, and 10 mg·L^−1^. The extraction recovery values ranged 80.1–98.8%, and the RSD values ranged 0.45–4.01%, as indicated in Table 1.

Satisfactory extraction recoveries were obtained for all targeted compounds, as well as at all three concentrations, with the values ranging from 80% to 98.8%. The method accuracy was evaluated by the RSD values of three replicates and was in the range of 1.08–3.66% at low concentrations, 1.04–4.56% at medium concentrations, and 0.45–4.01% at high concentrations, indicating favorable method accuracy. The reproducibility (interday precision) of the method is summarized in Table 2.

The methods yielded satisfactory results regarding both the intra- and interday precision levels of the analytical procedure, with RSD values lower than 15% (ICH acceptance criteria) for all the targeted compounds.

### 3.6. Stability Study

Establishing a set of conditions to ensure that a given analyte remains stable during transportation and storage without statistically significant concentration changes is an essential step in pharmaceutical compound analysis. Therefore, the study of the stability of target compounds is important. In the case of a certain process or analytical method, a stability study can provide evidence regarding how the concentration of a given analyte in a sample or container varies over time. This is influenced by a variety of environmental factors, such as temperature, storage conditions, and light exposure.

**Figure 3:**
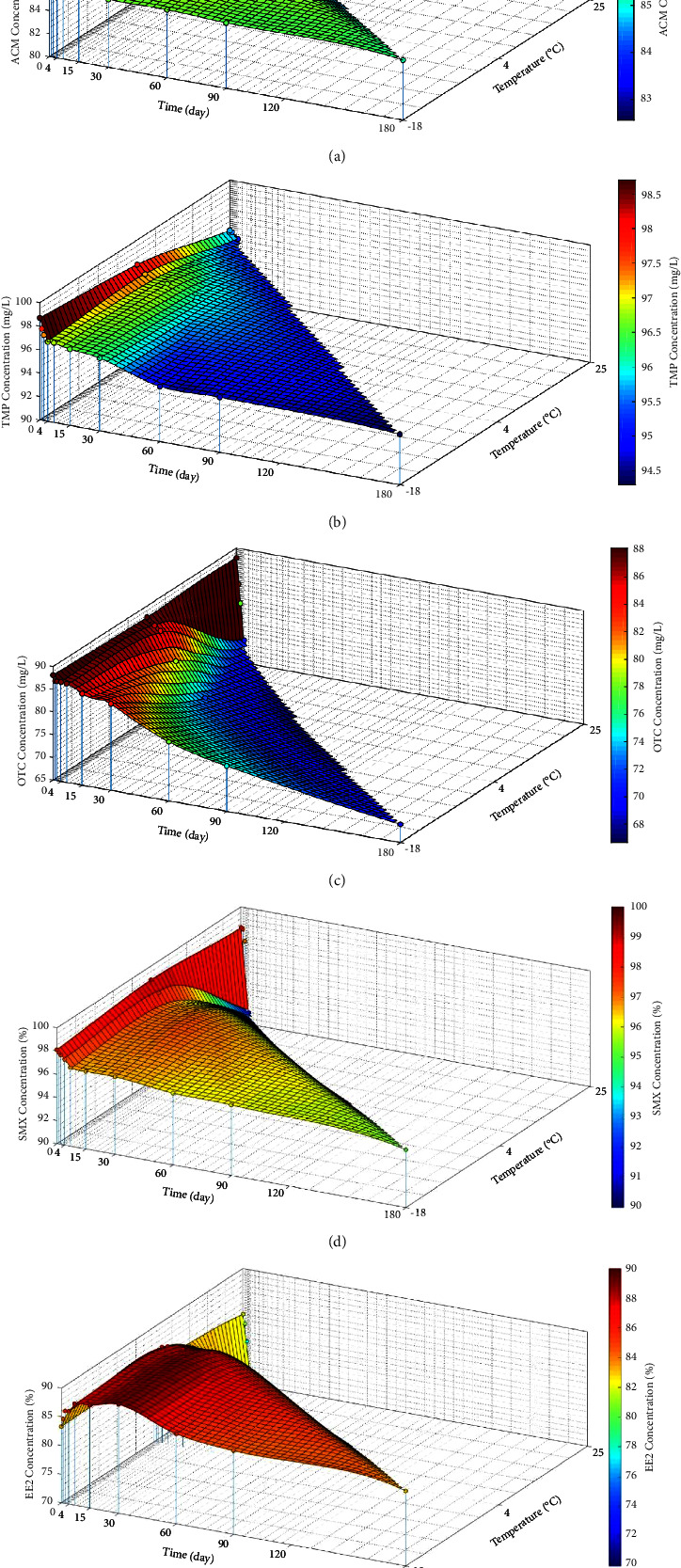
Stability of ACE (a), TMP (b), OTC (c), SMX (d), and EE2 (e) in SPE cartridges stored at −18, 4, and 25°C in the dark.

The stability of ACM, TMP, OTC, SMX, and EE2 in the SPE cartridge was evaluated after storage at −18°C, 4°C, and room temperature in the dark at various holding times, and the results are shown in Figure 3. Preventing light exposure is important for certain pharmaceuticals, such as OTC, since light exposure can cause faster photodecomposition than that under dark conditions [58]. It was found that almost all target pharmaceuticals, when protected from light, remained stable after 6 months of storage at −18°C, indicating no significant degradation.

Generally, pharmaceuticals and personal care products require lower-temperature storage conditions to maintain chemical integrity over long periods [43]. Liu et al. [59] reported that OTC remained stable for 3 months when stored at −18°C. Doi and Stoskopf [58] determined the kinetics of OTC degradation under the influence of various environmental factors, and the results indicated that low temperatures favored high OTC stability levels, while high temperatures accelerated OTC degradation. Thus, it could be concluded that the samples must be stored at −18°C to maintain the initial concentration.

In contrast, we found that sample freezing increased the 17-alpha-ethynilestradiol concentration over the initial concentration, i.e., from 8.3 to 9.3 mg·L^−1^ (12% of the initial value). Therefore, −18°C is the best storage temperature for the samples.

Examination of the samples stored at 4°C revealed that no major changes in the analyte concentration occurred within 1 month of storage among all of the target compounds that could explain the magnitude of loss of the analyte. Similarly, the authors in [58] found no change in the OTC concentration after 77 days at 4°C.

Figure 3(c) shows that ACM, TMP, OTC, SMX, and EE2 could be safely stored at room temperature for 3 days since there was no degradation indication (<15% change in the initial concentration). The authors in [58] reported that OTC remained thermolabile at 43°C, and in contrast, OTC exhibited a significantly higher stability at 4 and 25°C. The authors in [59] reported that half of OTC was degraded when stored at room temperature for 1 week.

## 4. Conclusion

Stability studies provide insights into the duration pharmaceutical compounds can maintain their concentration under varying environmental factors before analysis. Our study suggests that ACM, TMP, SMX, OTC, and EE2 remain stable when stored for 6 months at −18°C, protected from light. Similarly, stability is maintained for 1 month at 4°C. At room temperature, ACM, TMP, SMX, OTC, and EE2 can be stored for up to 3 days without significant degradation. However, it is important to note that freezing at −18°C did not result in significant changes in most compounds, except for EE2. In summary, the recommended conditions for environmental sampling are as follows: (a) field sampling and storage conditions: in the dark at ≤4°C. (b) Transportation and storage conditions: in the dark, for a maximum of 2 days at room temperature and/or ≤4°C. (c) Holding times: in the dark, for a maximum of 1 month at 4°C or frozen at −18°C for long-term storage.

It is worth noting that our study did not investigate the effects of freeze-thaw cycles, which could be a consideration for future research.

## Figures and Tables

**Table 1:** Recovery of extraction (RE, %) of the target compounds obtained during the repeatability (intraday precision) test. Average of three replicate SPE extractions of a spiked artificial seawater sample.

Target compounds	5 mg·L^−1^		7.5 mg·L^−1^		10 mg·L^−1^	
	RE (%)	RSD (%)	RE (%)	RSD (%)	RE (%)	RSD (%)
ACM	84.2 ± 3.0	3.7	88.2 ± 3.5	3.9	85.3 ± 0.9	1.0
TMP	95.5 ± 3.3	3.4	94.4 ± 0.9	1.0	98.8 ± 0.4	0.4
OTC	88 ± 1.5	1.7	89.3 ± 2.7	3.0	87.8 ± 1.1	1.3
SMX	97.1 ± 1.1	1.1	96.3 ± 2.1	2.1	97.1 ± 0.8	0.9
EE2	81.8 ± 2.1	2.6	85.9 ± 3.9	4.6	80.1 ± 3.2	4.0

**Table 2:** Evaluation of the intraday and interday precision (reproducibility) levels of the method.

Compounds of interest	Intraday (*n* = 6)		Interday (*n* = 12)	
	Mean concentration ± SD (mg·L^−1^)	RSD (%)	Mean concentration ± SD (mg·L^−1^)	RSD (%)
ACM	8.5 ± 0.1	1.28	8.4 ± 0.3	3.31
TMP	9.8 ± 0.2	1.28	9.9 ± 0.2	1.99
OTC	8.7 ± 0.1	1.39	8.4 ± 0.6	7.12
SMX	9.7 ± 0.1	0.78	9.7 ± 0.2	1.83
EE2	8.1 ± 0.3	3.43	8.0 ± 0.2	2.94

## Data Availability

The data that support the findings of this study are available from the corresponding author upon written request.
